# Head and Maxillofacial Injuries in Child and Adolescent Victims of Automotive Accidents

**DOI:** 10.1155/2014/632720

**Published:** 2014-12-10

**Authors:** Alessandro Leite Cavalcanti, Thiago Henrique de Araujo Lino, Thaliny Batista Sarmento de Oliveira, Thaisy Sarmento Batista de Oliveira, Andreia Medeiros Rodrigues Cardoso, Rodrigo Feliciano de Macedo, Wilton Wilney Nascimento Padilha, Alidianne Fabia Cabral Xavier

**Affiliations:** ^1^Post Graduate Program in Public Health, Centro de Integração Acadêmica, State University of Paraiba, Avenida das Baraúnas 351, 3° Andar/Sala 310, Campus Universitário, S/N, Bodocongo, 58429-500 Campina Grande, PB, Brazil; ^2^Department of Dentistry, Faculty of Dentistry, State University of Paraiba, Avenida das Baraúnas, S/N, Bodocongo, 58429-500 Campina Grande, PB, Brazil; ^3^Department of Dentistry, Faculty of Dentistry, Federal University of Paraiba, Cidade Universitaria, Castelo Branco, 58051-900 Joao Pessoa, PB, Brazil

## Abstract

*Background*. Victims of motor vehicle accidents may suffer multiple lesions, including maxillofacial injuries. The aim of this study was to evaluate the prevalence and factors associated with head, facial, and maxillofacial injuries in child and adolescent victims of automobile accidents. A cross-sectional study was carried out with analysis of forensic medical reports from the Legal Medical Institute of Campina Grande, Brazil, between January 2008 and December 2011. Descriptive and inferential statistical analysis was conducted using the chi-square test (*α* = 0.05). From 1613 medical reports analyzed, the sample is composed 232 (14.4%) reports referring to child and adolescent victims of automobile accidents aged 0–19 years of both sexes. Victims were mostly adolescents aged from 15 to 19 years (64.2%), males (73.7%), and motorcyclists (51.3%). More than half of the victims had single lesions (54.3%) located in the head (20.7%) and face (21.6%). Head injuries occurred more frequently in children aged 0–4 years (53.8%, PR = 5.065, 95% CI = 1.617–5.870) and pedestrians (30.4%, PR = 2.039, 95% CI = 1.024–4.061), while facial and maxillofacial injuries occurred in higher proportion among females (31.1%, PR = 0.489, 95% CI = 0.251–0.954). Our findings suggest that accidents involving motorcyclists are the most prevalent, affecting male adolescents aged from 15 to 19 years, resulting in a high frequency of injuries in the head and face regions.

## 1. Introduction

Unintentional injuries are the leading causes of morbidity and mortality in children and adolescents [1]. Individuals in this stage of life are looking for new references and experiences, resulting in risky behavior and exposure to certain injuries [2].

Within the context of unintentional injuries, the head region constitutes the most affected area in pediatric patients [3–5], and these injuries can be associated with severe temporary or permanent consequences, and they are responsible for almost 90% of all pediatric deaths [6]. Therefore, unintentional injuries are responsible for physical, emotional, social, and economic damage, including medical care expenses [3, 7], thus becoming a public health problem.

Automobile accidents are the major cause of unintentional maxillofacial and head injuries in the pediatric population [8, 9]. The prevalence of these lesions ranges from 34.2% [3] to 57.8% [10]. In Brazil, a previous study showed a frequency of 50% head injuries and 56.6% intraoral injuries among adolescent victims of automobile accidents [8]. Victims of motor vehicle accidents may suffer multiple lesions, including maxillofacial injuries [11–14].

However, few studies have focused on the factors associated with injuries in the head region, especially maxillofacial injuries in the pediatric population [5, 8, 9]. Studies have identified not only the factors involved in their occurrence, but also the social environment in which they occur, resulting in greater visibility [2]. Nevertheless, studies on this subject help to clarify the circumstances and support the development of clinical auditing, services management, and public policies for health prevention and promotion [9].

Given the above, this study aimed at determining the prevalence and factors associated with head, facial, and maxillofacial injuries in Brazilian children and adolescent victims of automotive accidents.

## 2. Materials and Methods

### 2.1. Design and Sample

A cross-sectional study design was undertaken through the analysis of expert medical reports derived from medical forensic exams performed at the Department of Forensic Medicine in the city of Campina Grande, PB, Brazil, between January 2008 and December 2011. The city of Campina Grande presented considerable cultural, social, and economic disparities, with an average monthly income of $110 per capita and a Human Development Index of 0.72.

From a universe of 1613 reports issued in this time span, the study sample consisted of 232 reports (14.4%) referring to children and adolescents of both sexes aged 0 to 19 years who had been victims of motor vehicle accidents.

### 2.2. Data Collection

Data referring to the victims' sex, age, day of week of accident, type of accident (pedestrian, cyclist, motorcycle, or occupant vehicle), number of existing injuries, presence of fractures, anatomic location of injuries (head: ICD-10 S01, face: ICD S09, maxillofacial: ICD-10 S02.9, and oral cavity: ICD S01.5), and presence of maxillofacial fractures (ICD-10 S02.4) were gathered from the forensic medical reports and transferred to specific registration forms, which were kept in folders classified according to event.

A road traffic injury was defined as any injury (regardless of severity) that occurred while walking, bicycling, or riding in a vehicle due to a crash involving one or more vehicles (including bicycles) and originating or terminating on a roadway [15].

### 2.3. Statistical Analysis

Data analysis involved descriptive statistics (frequency distribution) and analytic statistics. Bivariate analyses were conducted to test the association between the occurrence of head, face, and maxillofacial injuries and sex and age of the victims. This process was performed using the exact versions of the nonparametric Pearson's chi-squared test or Fisher's exact test. The prevalence ratios (the values obtained by dividing the prevalence of one category by the prevalence of another category of the same variable, e.g., prevalence of women divided by the prevalence of men) and their 95% confidence intervals were also calculated. The significance level established for all statistical analyses was 5% (*P* ≤ 0.05), and they were conducted using SPSS 18.0 (Statistical Package for the Social Sciences for Windows, SPSS Inc., Chicago, IL, USA).

### 2.4. Ethical Approval

This study followed ethical guidelines recommended by the Brazilian legislation and was approved by the Human Research Ethics Committee of the State University of Paraiba. All participants/guardians signed the informed consent form.

## 3. Results

Victims of automotive accidents were mostly adolescents aged 15–19 years (64.2%), mean of 14.67 (SD = 4.69) years, male (73.7%), and motorcyclists (51.3%) (Table 1). Male-to-female ratio was 2.8 : 1.

**Figure 1:**
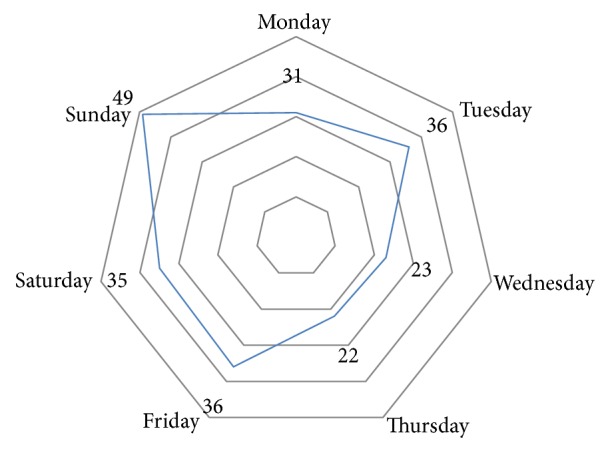
Distribution of accidents according to the day of the week in Campina Grande, Brazil.

Figure 1 shows the distribution of automotive accidents according to the day of the week, indicating that more than one-third of cases occurred over the weekend (36.2%), with predominance on Sunday (21.1%). The lowest prevalence was recorded for Thursday (9.5%).

Regarding the number of lesions, single lesions were the most frequent (54.3%). However, 65.9% had fractures in various regions of the body, and maxillofacial fractures were identified in only 2.6% of victims. To analyze the anatomical location, it was found that maxillofacial and head injuries occurred in 21.6% and 20.7%, respectively (Table 2). Only 5.6% of victims exhibited intraoral injuries.

Table 3 shows association between head, facial, and maxillofacial injuries and demographic and automotive accident characteristics. Association between presence of head injuries and age of the victim (*P* < 0.05) and between presence of head injuries and type of automotive accident (*P* < 0.05) was observed, with greater frequency among children aged 0–4 years (53.8%, PR = 5.065, 95% CI = 1.617–5.870), with such injuries being five times more frequent in this age group than among children aged 5–9 years, and greater frequency also among pedestrians (30.4%, PR = 2.039, 95% CI = 1.024–4.061). Regarding face injuries, only association with sex was observed (PR = 0.489, 95% CI = 0.251–0.954).

When analyzing accidents involving motorcyclists separately, there was an association between this type of accident and the occurrence of head injuries (*P* < 0.001, PR = 0.310, 95% CI = 0.156–0.616) and lower limbs (*P* < 0.05, PR = 1.765, 95% CI = 1.029–3.029). No association between this variable and the presence of face and upper limb injuries was found (*P* > 0.05). However, there was an association between motorcycle accidents and the presence of injuries (*P* < 0.05, PR = 1.789, 95% CI = 1.033–3.100).

## 4. Discussion

Unintentional head and maxillofacial injuries largely contribute to morbidity and mortality in pediatric and young adult populations [1, 5, 8, 14]. Social, cultural, and environmental factors have been associated with the pattern of head and maxillofacial injuries, especially those caused by automobile accidents [9, 12].

The present study, which focused on road traffic accidents, is probably one of the few studies developed in Brazil with the aim of investigating the characteristics of victims, especially those involving the head and face regions. These data are of particular importance to dentists and pediatricians.

The high morbidity and mortality rates related to transport accidents in Brazil have been associated with the fact that private cars are usually preferred over other means of transportation and that the roads are the main mode of circulation, even though they do not offer adequate conditions in terms of conservation and safety [16].

The analysis of gender and age distribution showed that the highest prevalence of road traffic accidents involved male victims aged 15 to 19 years, which is consistent with findings of previous studies [2, 3, 13, 17, 18]. This predominance illustrates the effect of sociocultural behavior, crystallized in the notion of sex and age, which could be explained by the fact that males take more risks when driving vehicles, experiencing feelings of risk, beyond the abuse of alcohol or drugs [2, 3, 17].

Regarding the type of automobile accident, those involving motorcycles were the most prevalent, as reported by other authors [2, 5, 8, 19]. The transportation of children and adolescents on motorcycles is a common practice in small- and medium-sized Brazilian cities such as Campina Grande, where the present study was conducted, because this is the main means of transportation among the low-socioeconomic-status population [5]. Other factors such as difficulty of visualization of motorcycles by other drivers, occurrence of inappropriate behavior in traffic, and disregard of traffic laws, beside the fact that few cities designate exclusive lanes for motorcyclists, stand out [19].

Also with respect to accidents involving motorcyclists, it is important to note that one-fifth of victims were female, which reinforces the hypothesis that women are more often using this means of transportation to perform daily activities [20].

Accidents involving pedestrians are the second major type of road traffic accidents. A pedestrian, with its relatively small mass compared to that of a motor vehicle, offers little resistance, absorbing the impact energy, which increases the morbidity and mortality rates for this group of victims [21].

As for the day of the week, regardless of type, most accidents occurred on Fridays and Sundays, with the weekend accounting for more than one-third of traffic accidents. The higher occurrence on weekends may be related to risky behavior in traffic such as driving over the speed limit, disrespect of traffic rules, and driving under the influence of alcohol.

Although more than half of victims have a single lesion, two-thirds of children and adolescents had injuries in different body regions. There was an association between being a victim of motorcycle accident and presenting head and lower limb injuries. Motorcycle drivers do not rely on an outer structure to protect them, absorbing most of the impact energy, and therefore are commonly thrown against the ground. These victims suffer, besides the impact from the accident itself, also the impact against the ground, usually followed by sliding [22].

The prevalence of head, facial, and maxillofacial injuries was similar to that reported by other studies [2, 9, 18]. Speed, position of victim, use of safety devices, and surface impact geometry are mostly responsible for the degree of impact and injuries sustained in road traffic accidents [23].

Some limitations of this study should be highlighted. It is possible that a small portion has never been to the Institute of Forensic Medicine to conduct the* corpus delicti *forensic examination as recommended by Brazilian legislation. Another limitation includes incomplete or missing data within the forensic medical record.

Considering the physical, psychological, and emotional distresses that accompany these injuries, it is important for our government to legislate and enforce traffic rules, strengthen road safety measures, and also implement poverty alleviation programs [23].

## 5. Conclusion

Accidents involving motorcyclists are the most prevalent, affecting male adolescents aged from 15 to 19 years, resulting in a high frequency of head and face injuries.

## Figures and Tables

**Table 1:** Sample distribution according to age, type of accident, and sex in Campina Grande, Brazil.

	Sex						
Variable	Male		Female		Ratio	Total	
	*n*	%	*n*	%		*n*	%
Age (years)							
0–4	8	61.5	5	38.5	1.6 : 1	13	5.6
5–9	16	66.6	8	33.4	2 : 1	24	10.4
10–14	35	76.1	11	23.9	3.2 : 1	46	19.8
15–19	112	75.2	37	24.8	3 : 1	149	64.2
Total	**171**	**73.7**	**61**	**26.3**	**2.8 : 1**	**232**	**100.0**
Victim of accident							
Pedestrian	39	69.6	17	30.4	2.3 : 1	56	24.1
Cyclist	5	100.0	0	0.0	—	5	2.2
Motorcycle	95	79.8	24	20.2	3.9 : 1	119	51.3
Occupant vehicle	30	60.0	20	40.0	1.5 : 1	50	21.6
Others	2	100.0	0	0.0	—	2	0.9

**Table 2:** Distribution of victims according to the number of lesions, existence of fracture, maxillofacial injury, and anatomical region involved in Campina Grande, Brazil.

Variable	Frequency	
	*n*	%
Number of lesions		
Single	126	54.3
Multiple	106	45.7
Fracture		
Yes	153	65.9
No	79	34.1
Maxillofacial injury		
Yes	6	2.6
No	226	97.4
Anatomical region		
Head	48	20.7
Face	50	21.6
Maxillofacial	50	21.6
Intraoral	13	5.6

**Table 3:** Association between sex, age, type of accident, and occurrence on weekends and the presence of head, face, and maxillofacial injuries in Campina Grande, Brazil.

Variable	Head injury		*P* value	PR (IC 95%)	Face injury		*P* value	PR (IC 95%)	Maxillofacial injury		*P* value	PR (IC 95%)
	Yes	No			Yes	No			Yes	No		
	*n* (%)	*n* (%)			*n* (%)	*n* (%)			*n* (%)	*n* (%)		
Sex												
Male	31 (18.1)	140 (81.9)			31 (18.1)	140 (81.9)			31 (18.1)	140 (81.9)		
Female	17 (27.9)	44 (72.1)	>0.05	0.573 (0.290–1.133)	19 (31.1)	42 (68.9)	<0.05	0.489 (0.251–0.954)	19 (31.1)	42 (68.9)	<0.05	0.489 (0.251–0.954)
Age												
0–4 years	7 (53.8)	6 (46.2)	<0.05	5.065 (1.617–5.870)	5 (38.5)	8 (61.5)	>0.05	2.417 (0.754–7.743)	5 (38.5)	8 (61.5)	>0.05	2.417 (0.754–7.743)
5–9 years	4 (16.7)	20 (83.3)		1.00	3 (12.5)	21 (87.5)		1.00	3 (12.5)	21 (87.5)		1.00
10–14 years	10 (21.7)	36 (78.3)		1.082 (0.493–2.374)	10 (21.7)	36 (78.3)		1.014 (0.463–2.219)	10 (21.7)	36 (78.3)		1.014 (0.463–2.219)
15–19 years	27 (18.1)	122 (83.9)		0.653 (0.342–1.248)	32 (31.5)	117 (78.5)		0.988 (0.514–1.896)	32 (31.5)	117 (78.5)		0.988 (0.514–1.896)
Type of accident												
Pedestrian	17 (30.4)	39 (69.6)	<0.01	2.039 (1.024–4.061)	12 (21.4)	44 (78.6)	>0.05	0.990 (0.476–2.060)	12 (21.4)	44 (78.6)	>0.05	0.990 (0.476–2.060)
Cyclist	2 (40.0)	3 (60.0)		2.623 (0.426–16.161)	1 (20.0)	4 (80.0)		0.908 (0.999–8.312)	1 (20.0)	4 (80.0)		0.908 (0.999–8.312)
Motorcycle	14 (11.8)	105 (88.2)		1.00	22 (18.5)	97 (81.5)		1.00	22 (18.5)	97 (81.5)		1.00
Occupant vehicle	15 (30.0)	35 (70.0)		1.935 (0.949–3.947)	15 (30.0)	35 (70.0)		1.800 (0.886–3.655)	15 (30.0)	35 (70.0)		1.800 (0.886–3.655)
Others	0 (0.0)	2 (100.0)		1.264 (1.183–1.350)	0 (0.0)	2 (100.0)		1.278 (1.194–1.368)	0 (0.0)	2 (100.0)		1.278 (1.194–1.368)
Weekend												
Yes	15 (17.9)	69 (82.1)	>0.05	0.758 (0.384–1.498)	23 (27.4)	61 (72.6)	>0.5	1.690 (0.895–3.191)	23 (27.4)	61 (72.6)	>0.05	1.690 (0.895–3.191)
No	33 (22.3)	115 (77.7)			27 (18.2)	121 (81.8)			27 (18.2)	121 (81.8)		
